# Efficient sensory cortical coding optimizes pursuit eye movements

**DOI:** 10.1038/ncomms12759

**Published:** 2016-09-09

**Authors:** Bing Liu, Matthew V. Macellaio, Leslie C. Osborne

**Affiliations:** 1 Department of Neurobiology, The University of Chicago, 947 East 58th Street, P415 MC0928, Chicago, Illinois 60637, USA; 2 Department of Organismal Biology and Anatomy, The University of Chicago, Chicago, Illinois 60637, USA

## Abstract

In the natural world, the statistics of sensory stimuli fluctuate across a wide range. In theory, the brain could maximize information recovery if sensory neurons adaptively rescale their sensitivity to the current range of inputs. Such adaptive coding has been observed in a variety of systems, but the premise that adaptation optimizes behaviour has not been tested. Here we show that adaptation in cortical sensory neurons maximizes information about visual motion in pursuit eye movements guided by that cortical activity. We find that gain adaptation drives a rapid (<100 ms) recovery of information after shifts in motion variance, because the neurons and behaviour rescale their sensitivity to motion fluctuations. Both neurons and pursuit rapidly adopt a response gain that maximizes motion information and minimizes tracking errors. Thus, efficient sensory coding is not simply an ideal standard but a description of real sensory computation that manifests in improved behavioural performance.

## Abstract (web summary)

Efficient coding suggests that adapting gain to match the varying stimulus statistics should help in optimizing behaviour. Here the authors show that adaptation in motion sensitive neurons maximizes information and improves movement accuracy in pursuit eye movements.

## Graphical abstract

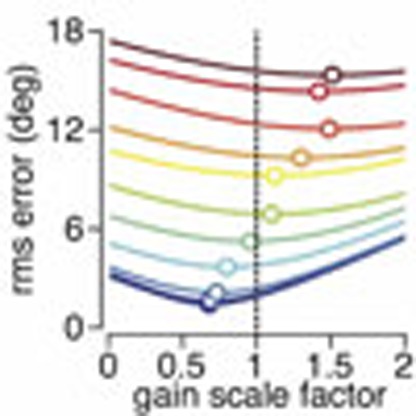

In a rapidly changing world, neural systems can optimize their representation of incoming stimuli by adjusting their sensitivity as stimulus conditions change^1^,^2^,^3^,^4^. As individual sensory neurons have a limited response bandwidth, how firing rates are allocated across the range of stimulus values affects how much information can be transmitted and, ultimately, how informative commands for behaviour can be^2^,^4^,^5^,^6^. When a signal varies little over time, a neuron can maximize its sensitivity by increasing its response gain, the change in firing rate per unit change in stimulus. When stimulus fluctuations grow large, lowering the gain avoids information loss from saturation. In theory, adaptation to variance, also known as temporal contrast, is an optimal coding strategy, because it allows individual sensory neurons to apply their full response bandwidth to encode incoming signals^2^,^3^. If neurons could maintain an optimal gain across changes in input statistics, the brain could theoretically recover more sensory information with which to guide behaviour. However, although the phenomenon of neural adaptation to input variance has been demonstrated^7^,^8^,^9^,^10^,^11^,^12^,^13^,^14^,^15^,^16^,^17^,^18^, its impact on information processing has only been reported in the fly visual system^3^,^5^,^6^ and the consequence for behaviour is unexplored^19^. To establish that gain adaptation at the neuronal level is important to the accuracy of sensory-motor behaviour, we have analysed the responses of sensory neurons and movement behaviour in parallel. Here we show that rapid gain adaptation to stimulus variance in visual cortical neurons optimizes information and movement accuracy in a primate oculomotor system.

In smooth pursuit behaviour, image motion on the retina is translated into a command to rotate the eye along with the target, to stabilize the retinal image^20^,^21^. Pursuit errors largely take the form of misestimates of target motion, which persist for ∼70–100 ms until visual feedback cues an alteration of the eye movement^21^,^22^. These errors result in image motion blur that degrades visual acuity, impacting perception and other visually driven behaviours^23^,^24^,^25^,^26^. Under natural conditions where target motion is dynamic, the quality of feed-forward visual estimates of target motion is critical to tracking acuity. The visual inputs for pursuit arise in cortical area MT (middle temporal area) where many neurons respond selectively to visual motion and responses are tuned for motion direction and speed^27^,^28^. In theory, MT neurons could maximize the information they transmit if they adjust their response gain such that their dynamic range spans as much of the range of current motion values as possible. Information savings at the level of individual neurons might in turn drive more accurate population-level motion estimates. For pursuit to benefit from an information savings at the cortical level, however, the adaptive gain changes must improve population motion estimates and must happen on the ∼70–100 ms timescale of the eyes' response to changes in target direction.

To determine whether pursuit behaviour displays the hallmarks of efficient coding, we measured the gain of the eye's response to fluctuations in target direction for different levels of overall direction variance. We performed a parallel set of experiments recording single units in MT to determine whether the behavioural effects had a cortical sensory origin. We find that both MT neurons, and pursuit behaviour as a whole, rapidly adopt a response gain that maximizes information about motion direction and minimizes tracking errors in pursuit. These data provide direct evidence of a functional benefit for efficient sensory coding.

## Results

### Experimental design

**Figure 1:**
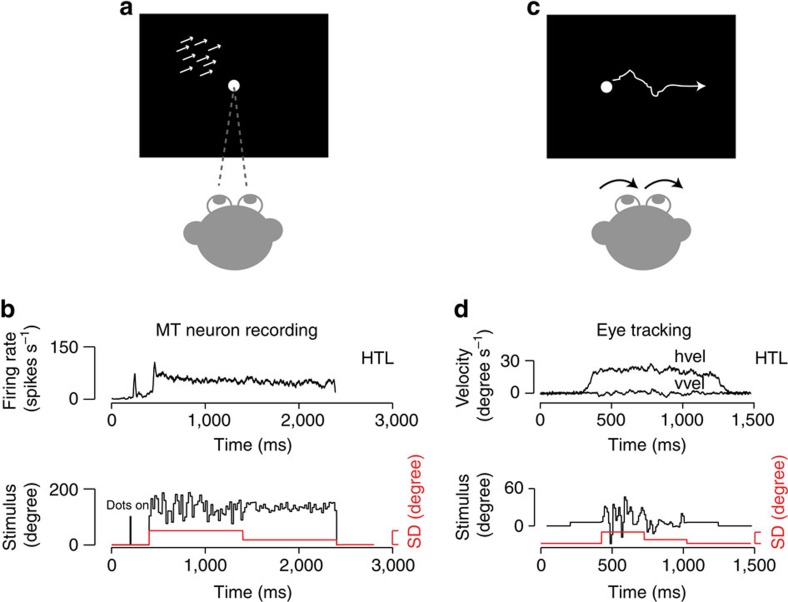
Experimental design and example data. (**a**) A fixation task kept motion stimuli centred on the receptive field. (**b**) Lower panel: stimuli had a constant drift speed and a mean direction chosen to fall on a flank of neuron's direction tuning curve. We added a stochastic direction perturbation, updated every 20 ms (black) that shifted from HTL (or LTH, not shown) variance during the trial (red). Upper panel: PSTH of an isolated MT neuron. (**c**) Pursuit task design. Targets translated across the screen with identical direction statistics as for **a** but in a randomly selected mean direction to minimize anticipation. (**d**) Upper panel: Horizontal and vertical components of eye velocity during a single trial with a HTL variance shift. Lower panel: target direction (black) or direction variance (red) over time.

**Figure 2:**
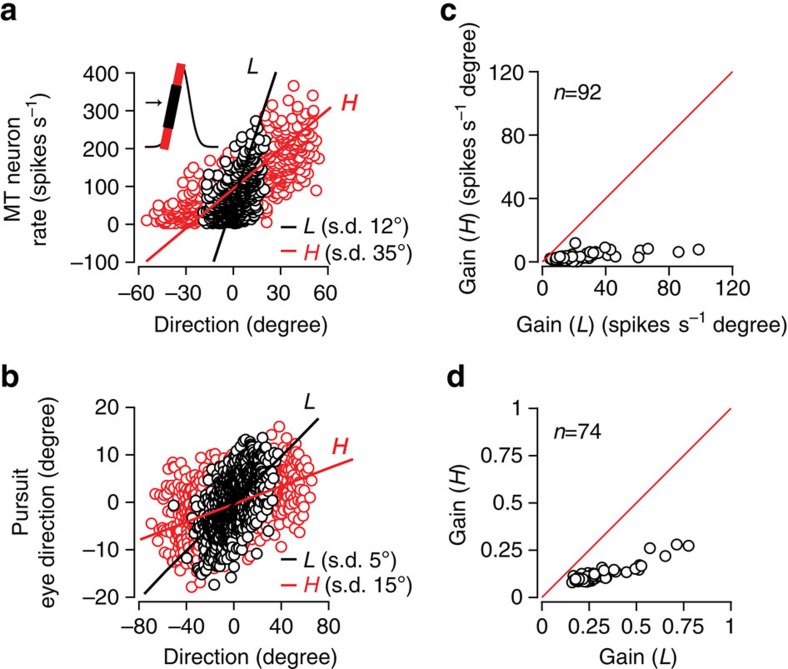
Gain rescales with stimulus variance in MT neurons and pursuit. (**a**) A single MT unit's input output function for a low-variance (*L*, s.d. 12°, black circles) and high-variance (s.d. 35°, red circles) motion stimulus. Circles represent mean values in 20 ms windows. Direction fluctuations measured with respect to the mean (black arrow, inset). Black, red lines correspond to the dominant mode of variation in the data and represent the best estimate of the response gain. (**b**) Eye movements from the same monkey: low variance (*L*, s.d. 5°, black circles) and high variance (*H*, s.d. 15°, red circles). (**c**) MT population data showing linear gain for *L* and *H* variance conditions in **a** (pop. mean *L*: 23±27; *H*: 3.0±2.1, significantly different, two-sided Wilcoxon rank-sum test, *P*<10^−28^, *n*=92). (**d**) Pursuit population data (pop. mean *L*: 0.28±0.13; *H*: 0.12±0.04, significantly different, two-sided Wilcoxon rank-sum test, *P*<10^−22^, *n*=74, 3 monkeys).

Our approach to testing for behaviourally relevant efficient sensory coding is inspired by natural pursuit behaviour, which is often called on to track targets with time-varying motion profiles, such as the flight path of an insect evading a fly swatter. We focus on direction fluctuations, creating motion stimuli that have a constant motion speed and time-averaged direction, with an added stochastic perturbation in direction. We performed two types of experiments, illustrated in Fig. 1 (see Methods). In the pursuit task, monkeys tracked targets that translated across the screen (Fig. 1c). In the fixation task, the same motion stimulus was presented within a stationary aperture centred over the receptive field of an MT unit, while we made extracellular recordings (Fig. 1a). In both cases, stimulus directions were randomly chosen from a uniform distribution every two frames (20 ms) and a new sequence was generated for each trial (Fig. 1b,d). We chose to separate MT recording from the pursuit experiments, to better control visual input for repeatability and to minimize complications from stimulus motion within the receptive field arising from eye movement. The bulk of our physiology data was collected such that the central direction of motion fell on the flank of each neuron's direction tuning curve and the direction range remained within the neuron's response range (Fig. 2a, inset). This configuration minimized changes in the time-averaged firing rate across step changes in direction variance, allowing us to isolate adaptive changes to direction variance. Our emphasis on adaptation in response gain (sensitivity to fluctuations) rather than magnitude (mean firing rate) distinguishes this work from ‘after-effect' studies using exposure to constant stimuli, to manipulate firing rates and tuning curves^29^,^30^,^31^,^32^,^33^.

For both the MT and pursuit experiments, trials were typically divided into two or more segments (100–2,000 ms) for which the time-averaged direction remained constant, but the direction variance stepped between lower and higher values, which we term LTH (low to high) or HTL (high to low), to indicate an upward or downward variance step, respectively (Fig. 1b,d). Although MT neurons showed a transient response to motion onset, variance steps rarely (1/87) elicited a second transient response. Rather, the time-averaged firing rate remained fairly constant (Fig. 1b). Other studies employing stimuli that alternately excited and inhibited spiking have reported rate transients after variance steps, for example, in the fly, salamander retina and cortical slices^5^,^6^,^7^,^8^,^11^,^12^,^13^,^14^,^15^,^34^,^35^,^36^,^37^,^38^. In contrast, our stimuli were configured to provide a time-varying firing rate without suppressing spiking altogether (see Fig. 2a). Despite the lack of a firing rate or eye velocity transient, we find that both MT neurons and pursuit shift their sensitivity to motion direction fluctuations after variance steps.

### Response gain rescales with stimulus variance

How an MT neuron or an eye movement responds to a motion fluctuation depends on context. The simplest illustration of the variance dependence of the neural (or behavioural) response is to plot the firing rate (or eye direction) versus the stimulus direction computed in short 20 ms time windows. The steady-state input–output relationships for a low- and high-variance direction stimulus are shown in Fig. 2. We have time shifted the stimulus and response values by the average response latency throughout. For the MT neuron, Fig. 2a represents a portion of the direction tuning curve. Symbol colour indicates low (black) or high (red) variance conditions. The slope of the linear fit represents the average change in response per unit change in stimulus and hence the gain, *g*=Δ*r*/Δ*s* (see Methods). For both MT neurons and pursuit, gain is high when direction variance is low and low when variance is high (Fig. 2a–d). We found this to be true across our cortical and behavioural samples.

**Figure 3:**
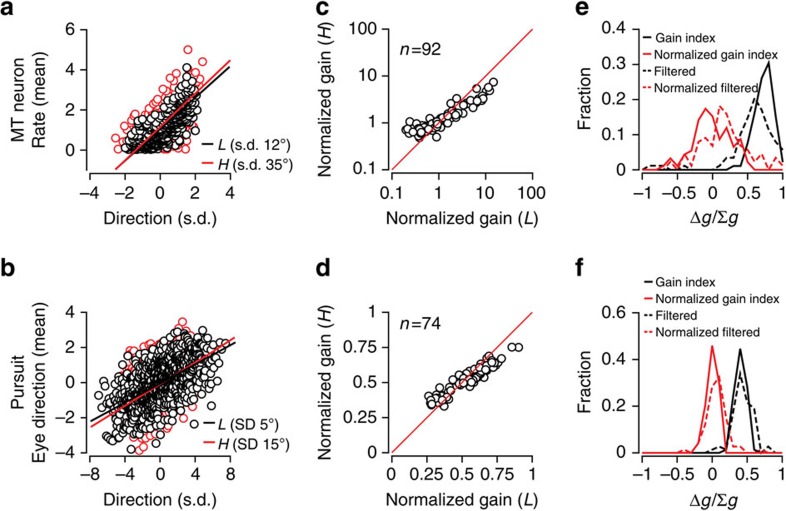
Adaptation normalizes response gain across variance conditions. (**a**) Plotting the stimulus in units of its s.d., the neuron's input–output relationship is the same for both variances (same data as Fig. 2a). (**b**) Rescaled input–output data from the same pursuit experiment. (**c**) MT population data, showing linear gain (red and black lines, **a**,**b**) recomputed for the normalized data (*L*: 2.2±2.9; *H*: 1.5±1.3, not significantly different, two-sided Wilcoxon rank-sum test, *P*=0.35, *n*=92). (**d**) Pursuit population data similar to **c** (*L*: 0.53±0.16; *H*: 0.52±0.10, not significantly different, two-sided Wilcoxon rank-sum test, *P*=0.61, *n*=74). (**e**) To analyse gain changes across the population, we defined a gain difference index (see text), Δ*g*/Σ*g*, where an index of 0 indicates perfect rescaling. We plot the distribution of MT neuron gain indices before (black solid line; pop mean±s.d.: 0.72±0.13, *n*=92, significantly different from 0, one-sample *t*-test, two-tailed, *P*<10^−30^) and after normalization (grey solid line; 0.01±0.26, *n*=92, not significantly different from 0, one-sample *t*-test, two-tailed, *P*=0.67). Low-pass-filtered motion stimuli (see text) yielded similar results (dashed lines). (**f**) Pursuit population data as in **e**: original data (black solid line, mean±s.d.: 0.40±0.08, *n*=74, 3 monkeys, significantly different from 0, one-sample *t*-test, two-tailed, *P*<10^−30^) and the rescaled input–output relationship (grey solid line, mean±s.d.: 0±0.07, *n*=74, 3 monkeys, not significantly different from 0, one-sample *t*-test, two-tailed, *P*=0.68). Low-pass-filtered stimuli again yielded similar results (dashed lines).

Rescaling response gain with stimulus variance can maximize information transmission^5^. If cortical and behaviour response gain compensates perfectly for changes in stimulus variance, then if we express the stimulus in units of its s.d., the gain values we compute across variance levels should coincide. We find this to be the case for both MT neurons and pursuit behaviour. Re-plotting the example data in Fig. 2 in units of the direction s.d. for both low (*L*) and high (*H*) variance levels, we find close agreement between the fitted gain values (Fig. 3a,b). Looking across the cortical and behavioural samples, we also find that the s.d.-normalized gain values for *L* and *H* high-variance conditions are very similar, plotting near the unity line in Fig. 3c,d (compare with Fig. 2c,d). For the MT sample (Fig. 3c), the s.d.-normalized *L* variance gain (2.2±2.9, mean±s.d.) was not significantly different from the s.d.-normalized *H* variance gain (1.5±1.3; two-sided Wilcoxon rank-sum test, *P*=0.35, *n*=92). The nine neurons in the sample with the highest firing rates and, therefore, the largest gain normalization factors appear to deviate from the linear relationship at the lowest normalized gain values (Fig. 3c). Despite the apparent curvature in neural gain scaling, a second-order polynomial fit accounts for only 3% more of the variance than a linear fit (*R*^2^=0.86 linear fit; *R*^2^=0.89 second-degree polynomial fit). For pursuit, the normalized gains at high versus low variance levels were quite similar (Fig. 3d): low gain: 0.53±0.16; high gain: 0.52±0.10, not significantly different, two-sided Wilcoxon rank-sum test, *P*=0.61, *n*=74 from 3 monkeys). Thus, the response gain shifts to compensate for changes in stimulus s.d. nearly perfectly on average.

To quantify the extent of gain adaptation in MT neurons and pursuit behaviour, we created an index, Δ*g*/Σ*g*, to capture the relative gain differences between low versus high variance conditions, that is, Δ*g*, in units of the sum, Σ*g*=(*g*_L_*+g*_H_). The gain indices were distributed ∼0.72±0.13 (*n*=92) for MT neurons and 0.4±0.08 (*n*=74) for pursuit, indicating that response gain is strongly variance dependent. However, the s.d.-scaled gain index for MT neurons and pursuit had an average value not statistically different from zero (neurons Fig. 3e, solid red line, one-sample *t*-test, two-tailed, *P*=0.67; pursuit Fig. 3f, solid red line, one-sample *t*-test, two-tailed, *P*=0.68), indicating perfect gain rescaling on average across our data samples.

Neither the neurons nor the behaviour showed a large difference in response ranges for the stimulus variance levels we tested. Rather, the gain shift appears to arise from a remapping of the response bandwidth onto the current range of direction inputs, potentially creating ambiguity in single-neuron direction coding arising from the lack of a fixed relationship between input and response, but maximizing sensitivity to direction changes^6^. To ensure that the invariance in the response range did not arise from saturation, we performed a control. The high (*H*) variance stimulus has more high-frequency power than the low (*L*) variance condition. If the system is insensitive to higher frequencies, then saturation might result in an apparent gain change without actual adaptation in the system^39^,^40^. We used the response bandwidth of pursuit, essentially a low-pass filter with a corner frequency of 20 Hz, to filter out higher-frequency components of the stimulus and then recalculated the gain values for *L* and *H* conditions. If saturation to high-frequency components were masquerading as a gain shift, the filtered *L* and *H* stimuli would yield similar gain values. Rather, we found that the gain values changed very little with the filtered stimuli. The gain index values were positive for both the broadband (black solid lines: neurons Fig. 3e, mean±s.d. 0.72±0.13, *n*=92; pursuit Fig. 3f, 0.40±0.08, *n*=74) and low-pass filtered stimuli (black dashed lines: neurons Fig. 3e, 0.56±0.34, *n*=88; pursuit Fig. 3f, 0.44±0.14, *n*=74). Normalizing by the stimulus, s.d. shifted the gain index distribution to a near zero mean, indicating that the response gain scaled with stimulus variance, similarly for both the broadband (grey solid lines: neurons Fig. 3e, 0.01±0.26, *n*=92; pursuit Fig. 3f, 0±0.07, *n*=74) and low-pass stimuli (grey dashed lines: neurons Fig. 3e, 0.17±0.33, *n*=88; pursuit Fig. 3f, 0.07±0.15, *n*=74).

### Adaptation maximizes information in MT and pursuit

**Figure 4:**
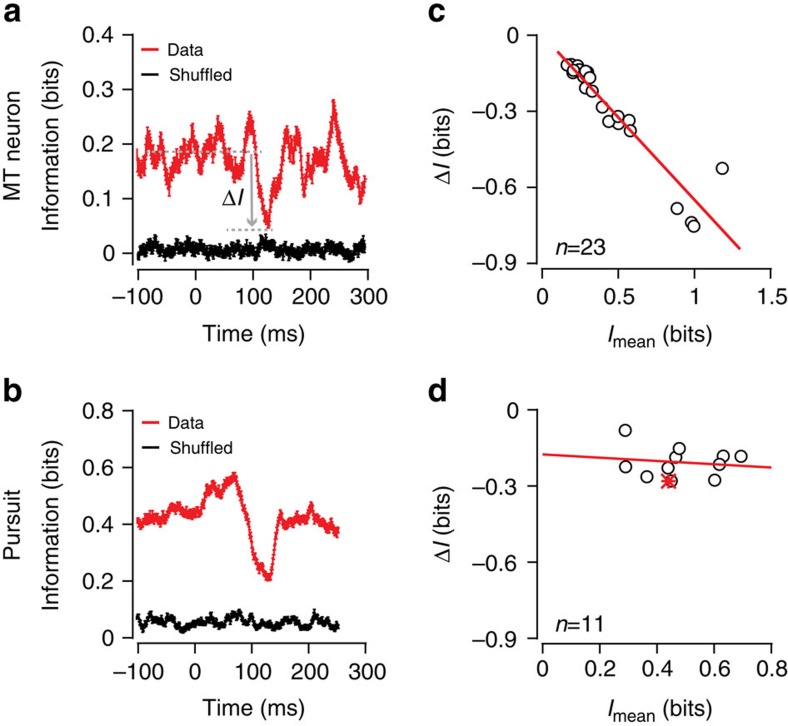
Rapid recovery of mutual information after variance step. (**a**) Red trace: mutual information between stimulus direction and spike count (firing rate) for a single MT neuron computed in a sliding 60 ms time window across a HTL variance step (s.d.=35° to 12°); (black trace) shuffled data. (**b**) Mutual information between eye and target direction from a single data set across an HTL variance shift (s.d.=15 to 5°). We plotted Δ*I*, the difference between the minimum information value at the dip and the time-averaged information level before the dip, that is, <*I*>, against <*I*> for the (**c**) MT data (*n*=23) and (**d**) pursuit data (*n*=11, 3 monkeys). Red lines represent linear regressions. For MT, the information dip was a constant fraction of the average information value (slope=−0.65, *R*^2^=0.88, *n*=23). For pursuit, the dip was less dependent on the information level, 0.21±0.06 bits (slope=−0.06, *R*^2^=0.02, *n*=11). The red star in **d** indicates the simulated neural population prediction for pursuit, which is quite close to the observed data (see text).

Adaptation is beneficial for perception and movement if it serves to maximize information about the incoming signal. To quantify the impact of gain adaptation on sensory coding, we computed the mutual information between spike counts and stimulus directions over time across steps in direction variance. The advantage of information theory is that it yields a model-independent result that incorporates any response nonlinearities^41^. We estimated the joint stimulus-response distributions (stimulus direction versus spike counts or binned eye direction) in overlapping 60 ms time windows (see Methods). The probability of observing stimulus-response pairings is a function of the temporal separation between the stimulus and response windows. Being careful to correct for sampling bias (see Methods)^42^,^43^, we then estimated the mutual information at each time delay between the stimulus and response, as a function of time within the trial. As expected, the delay that maximized information corresponded to the time to peak of the spike-triggered average stimulus. Similarly, the optimal delay for pursuit corresponded to the response latency. As shown in Fig. 4a,b, information about motion direction is constant throughout the trial until just after the direction variance step. When the neuron begins to respond to the new direction distribution, the information dips but then rapidly recovers as the system adapts (Fig. 4a). The information recovery occurs after the neurons have fired, on average, 3.8±2.5 spikes (*n*=23) in response to the new variance condition. As with MT neurons, the mutual information between the eye and target direction shows a drop and then a rapid recovery after a step in direction variance (Fig. 4b). The similarity in neural and behavioural information recovery suggests that adaptation at the neural level allows the system as a whole to maintain performance across shifts in motion statistics. Although the average level of encoded information, *I*, differed across our cortical sample, the percentage drop after a variance step was roughly consistent for all neurons such that Δ*I=α × I*, where *α* is −0.65 by linear regression (*R*^2^=0.88, *n*=23; Fig. 4c). As visual estimates for pursuit arise from a population of MT neurons^43^, it is perhaps not surprising that we see less variable information levels in pursuit behaviour. In pursuit, the scale of the dip is not strongly dependent on the average level of information, with *α*=−0.06 (Fig. 4d, linear regression, *R*^2^=0.02, *n*=11). To determine whether the similarity of Δ*I* values in pursuit could be explained by the MT data, we simulated a population response by averaging the information time courses across units, plotting the resultant mean and dip value in Fig. 4d (red star). Although our neural sample is modest, there is close agreement between the predicted population response and pursuit data. Units with diverse tuning will contribute to behaviour, but our MT sample represents a subpopulation with maximal sensitivity to direction fluctuations and thus might contribute most strongly to behaviour^44^, explaining the result. We did not observe information dips after motion segment breaks without variance changes^6^.

**Figure 5:**
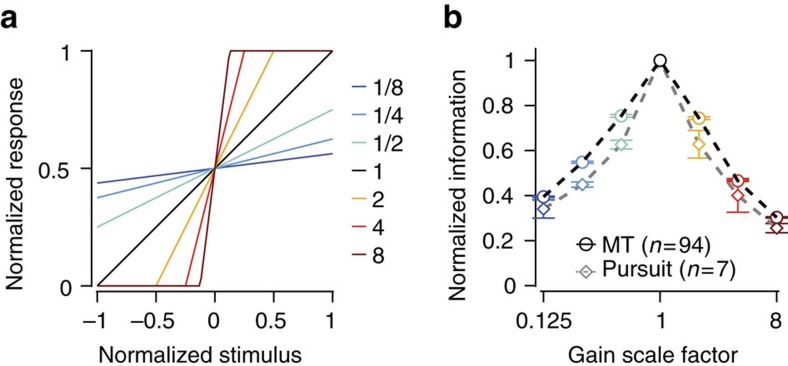
Adaptation maximizes motion information in MT and pursuit. (**a**) We artificially rescaled the response gain by a multiplicative scale factor from 1/8 to 8 (coloured lines) and re-computed the steady-state mutual information between neural (or pursuit) response and motion direction (see text). (**b**) The mutual information was lower for all scale factors other than 1 (unscaled data) across our sample. Population data for MT neurons (black line, *n*=94) and pursuit (grey line, *n*=7, 3 monkeys). Error bars are defined as s.d.

If the goal of adaptation is to optimize internal motion estimates and thereby motion-driven behaviours such as pursuit, then altering the response gain of either MT neurons or pursuit behaviour should degrade the mutual information between stimulus and response. We tested this hypothesis by analysing the dependence of the steady-state information on the response gain. By numerically rescaling the response relative to the stimulus, we simulated different gain levels (Fig. 5a) and recomputed the information for each level (Fig. 5b)^5^. We found that the true response gain (represented by the black line, Fig. 5a) always maximized direction information, across our cortical and behavioural data sets. This indicates that the information savings by encoding motion efficiently at the cortical level is reflected in ideal behaviour.

### Gain adaptation minimizes pursuit errors

**Figure 6:**
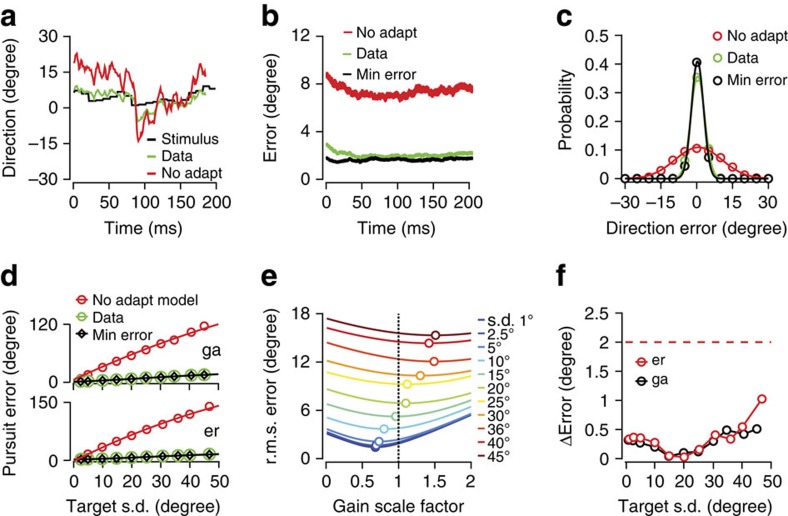
Adaptation optimizes pursuit by minimizing tracking errors. (**a**) Example pursuit trial showing target (black) and eye (green) over time for an s.d.=2.5° experiment. The simulated pursuit response without adaptation (red line, see text) is much less accurate. We have subtracted the average latency to align the target and eye data for visualization. (**b**) Trial-averaged direction errors from the experiment and simulation in **a**: pursuit (‘data', green, stimulus s.d.=2.5°, gain=0.7) has smaller direction errors than simulated fixed-gain pursuit (‘no adapt', red, gain=1.7). The minimum error level from a multiplicative rescaling of pursuit data is very close to the data itself (‘min error', black, gain=0.5). (**c**) Distribution of tracking errors at each millisecond from the same data (green, s.d._err_=2.5°) and simulation: non-adapting (red, s.d._err_=9.0°), min. error, (black, s.d._err_=2.0°). (**d**) Tracking errors across target variance levels: pursuit data (green), a fixed-gain (no adaptation) simulation (red) and the minimum error achievable from rescaling gain (black). Plots represent mean values over ten data sets for each monkey. (**e**) Error curves generated from the rescaling simulation (coloured lines). We rescaled the eye movement at each time step to simulate different response gains (from 0.1 to 2 times the actual pursuit gain), then computed the expected error *θ*_err_(*t)= θ*_targ_*(t−τ)*-scale_factor*θ_eye_*(t).* The r.m.s. error level is a convex function of the gain scale factor. Circles indicate minima. The dotted black line corresponds to actual pursuit gain and tracking errors at each variance level (data form monkey er). (**f**) The differences between minimum and actual tracking errors as a function of target direction s.d. for two monkeys (er, red; ga, black). The differences are well below the perceptual threshold for direction discrimination (red line, see text).

Performance in a tracking behaviour such as pursuit is defined by its accuracy: how well the eye movement follows target movement over time. If gain adaptation optimizes pursuit, then suppressing that adaptation should lower the tracking accuracy. We simulated a non-adaptive pursuit system by re-scaling the eye and target motions to manipulate the response gain, holding it fixed across a range of motion variance levels. For example, the gain value (*G*) measured for a stimulus with a direction variance s.d._T_=1° is approximate by the ratio *G*_1_°≈s.d._E_/s.d._T=_1.7, where the *T* and *E* subscripts represent the target and eye direction, respectively, and the s.d. describes fluctuations over time. If direction variance increases to s.d._T_=2.5° (black trace, Fig. 6a), the pursuit gain adapts to a lower value, *G*_2.5_°=0.7 (green trace, Fig. 6a). We simulated fixed-gain (non-adaptive) pursuit by multiplying the eye direction at each time point by a factor *G*_1_°/*G*_2.5_°=2.4 such that the gain remained 1.7 (red trace, Fig. 6a). It is apparent in Fig. 6a–c that tracking errors increase substantially without adaptation. We define tracking errors in the two-dimensional plane as the difference between the eye and target direction at each time step, 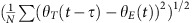, where *θ* represents the eye or target direction, *τ* the behavioural latency from the movement-triggered average stimulus and *N* the number of time steps across all trials. In the example data shown in Fig. 6a–c, tracking errors with adaptation (data) have a root mean squared (r.m.s.) value of 2.5° (green lines, Fig. 6a–c), whereas non-adaptive pursuit has much larger errors, s.d._err_=9.0° (significantly different, paired-sample *t*-test, two-tailed, *P*<10^−30^, *n*=20; red lines, Fig. 6a–c). The negative impact of suppressing adaptation increases with target direction variance (red versus green lines, Fig. 6d), such that r.m.s. tracking errors increase more than sevenfold for monkey Er (significantly different, paired-sample *t*-test, two-tailed, *P*<10^−3^, *n*=10) and more than ninefold for monkey Ga (significantly different, paired-sample *t*-test, two-tailed, *P*<10^−3^, *n*=10) compared with (adaptive) data.

The simulations we performed are realistic, because pursuit at different target direction variance levels is well described by a gain rescaling. The difference between tracking errors in simulated (that is, rescaled) versus actual data were small (∼0.2* s.d._E_) compared with the inherent variation in pursuit (difference between simulated pursuit and data: mean=0.02°, s.d.=0.58°, *n*=56)^22^,^45^,^46^.

Gain adaptation minimizes tracking errors across all direction variance conditions. We used the re-scaling approach to relate gain to r.m.s. direction errors for each target variance condition (coloured lines, Fig. 6e). By rescaling the eye relative to the target direction, we simulated lower and higher gain values, measuring the r.m.s. tracking errors for each gain scale factor. The error surfaces are concave and thus have a minimum value (coloured lines, Fig. 6e). The minimum error values (open circles) lie close to the errors observed in pursuit data (intersection of the dashed line with each curve, Fig. 6e). The deviations from the minimum values are small compared with the discrimination threshold for direction fluctuations (red dashed line, Fig. 6f)^22^,^45^,^46^, suggesting that the system is in fact minimizing tracking errors within the constraint of its natural variability.

### Dynamics of cortical and behavioural gain adaptation

The dynamics of an adaptation process can be suggestive of the underlying mechanism. For example, adaptation driven by changes in a channel conductance can be slow, with a time constant of seconds or longer^6^,^7^,^8^,^9^,^14^,^15^, whereas cortical synaptic facilitation/depression occurs more quickly, with time constants of ∼30–100 ms (ref. 47). To resolve the time at which a variance shift can be reliably detected from the response on each trial, we used a change-point detection method^48^. Change-point detection simulates an ideal observer who knows the distribution of responses under the two different stimulus conditions and steps through each time point within a trial to evaluate the likelihood that the current response arises from the *L* versus *H* conditions^48^,^49^ (see Methods). As the direction perturbation sequence on each trial is randomly generated and the responses are variable, the time at which the variance step can be detected differs from trial to trial. We found that detection times for LTH were shorter than for HTL transitions for both neurons and behaviour (MT neurons: *P*<10^−30^, *n*=13,623; pursuit: *P*<10^−30^, *n*=6,135, two-sided Wilcoxon rank-sum test). With respect to their average latencies, MT neurons detect upward (LTH) variance steps after 45±50 ms (*n*=13,623, 34 neurons) and downward (HTL) steps after 61±30 ms (*n*=13,745 trials). Pursuit responds to variance shifts slightly later: 53±44 ms (*n*=6,135 trials, 9 data sets) for LTH and 71±38 ms (*n*=6,597) for HTL variance transitions, again measured from response latency. These times are quite close to the earliest possible detection times, based on a statistical analysis of the stimulus on each trial (see Methods). MT neurons detect variance steps on average 1–2 ms after an ideal observer with complete knowledge of the stimulus distributions could (LTH 0.53±5.9 ms, *n*=13,623 trials; HTL, 2.2±8.2 ms, *n*=13,745 trials, 34 neurons) and pursuit 4–6 ms after (LTH 3.9±14.9 ms, *n*=5,664 trials; HTL 6.1±15.6 ms, *n*=5,977 trials, 9 data sets). The difference in detection times for upward versus downward variance steps is expected, because small direction perturbations are nearly as likely to arise from either distribution, whereas a large direction fluctuation immediately identifies an increase in variance. The rapidity of rescaling to upward variance transitions may be why we do not observe an information transient for LTH steps—essentially the transients are too narrow to detect^5^,^6^.

### Gain shifts depend on the experienced direction sequence

**Figure 7:**
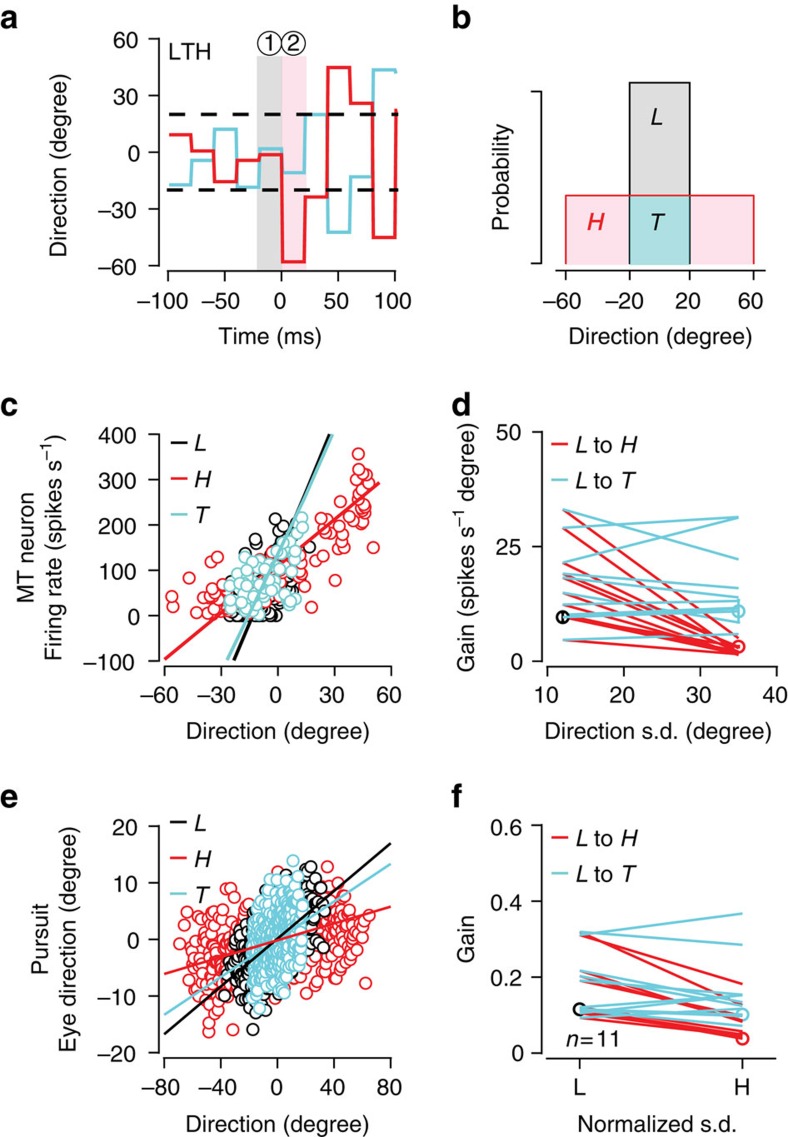
Gain adaptation depends on experienced stimulus values. (**a**) Using an example neuron, we analysed data just before (‘1', grey shading) and after (‘2', pink shading) a variance step (LTH, s.d.=12° to s.d.=35°) to determine how shifts in response gain depended on the actual stimulus direction sequence. (**b**) We separated trials based on whether time bin 2 direction values fell in the area of overlap between the *L* and *H* stimulus distributions (*T*, cyan shading). (**c**) Stimulus and response values in ‘bin 1' (*L*, black circles) and ‘bin 2' (*H*, red circles) for all trials. We have highlighted data from the ambiguous *T* subset of trials (‘bin 2 *T*', cyan circles). Linear gain values computed as in text (lines). (**d**) MT population data (*n*=11), gains measured in time bin 1 and time bin 2 on all trials (red) or only on ambiguous T trials (cyan). (**e**,**f**) Same as **c**,**d**, but for pursuit behavioural data. The response gain rescales with stimulus variance only if the animal sees an outlier value (*n*=11).

If the shifts in response gain do arise from adaptation, they should occur only after observing a stimulus outlier. The likelihood of observing a large direction change goes up after an upward variance transition, but in any random sequence the time at which the first outlier appears will vary from trial to trial. As a control, we selected the subset of trials from an upward transition experiment in which the first stimulus direction generated after the transition from LTH variance (time bin 2, Fig. 7a) had a value that could have arisen from the low direction variance distribution (cyan trace Fig. 7a; cyan area Fig. 7b). We then compared the gain state measured from those trials with the gain state at the preceding time step (time window 1, Fig. 7a). The data in Fig. 7c,e show the responses of an example neuron and behavioural data set for the time windows and stimulus distributions indicated in Fig. 7a,b. The best linear fit for the response gain for the ambiguous T trials (cyan, Fig. 7c,e) was statistically indistinguishable from the preceding *L* variance response gain (black, Fig. 7c,e) and quite different from the post-step (*H*) gain measured across all trials (red, Fig. 7c,e). We found no difference between the *L* variance response gain and the ambiguous (*T*) trial post-step gain across our cortical and behavioural data samples (MT neurons Fig. 7d, 22±15 for *L* and 17±10 for *T*, *P*=0.24, paired-sample *t*-test, two-tailed, *n*=11 neurons; pursuit Fig. 7f, 0.17±0.08 for *L* and 0.16±0.09 for *T*, paired-sample *t*-test, two-tailed, *P*=0.6, *n*=11 data sets). These results allow us to confirm that the gain change we observe is causally related to the experienced stimulus.

## Discussion

The theory of efficient coding is linked to the idea that neural systems maximize information relevant to behavioural performance that can influence survival^1^,^2^,^3^,^4^. Observations of neural responses in many organisms have demonstrated a capacity for efficient coding^7^,^8^,^9^,^10^,^11^,^12^,^13^,^14^,^15^,^16^,^17^,^18^, but the consequences for motor behaviour have not been explored^19^,^50^. These experiments break new ground, because they demonstrate that the principle of efficient coding applies to a neural system as a whole, improving the accuracy of the movements it generates and not solely to individual sensory neurons. We have exploited the close connection between cortical motion estimates and smooth pursuit eye movements^22^,^45^,^46^,^51^,^52^,^53^,^54^, to demonstrate parallel adaptation effects in sensory neurons and movement behaviour. Our experimental design separated the physiological and behavioural recording, to create the controlled repetition necessary to measure information in single neurons. Although this design does mean that we cannot directly relate fluctuations in each neuron's rate to fluctuations in pursuit, the fact that we observed parallel gain optimization in both neurons and behaviour encourages us to think that the adaptation we describe is a robust feature of sensory function. Adaptation is a broad concept that might include any modulation in firing rate. Here we specifically ask about adaptation to stimulus variance—a statistical feature of the environment—rather than to stimulus exposure per se such as studies of the motion after effect^29^,^30^,^31^,^32^,^33^. We find that adaptation to motion variance optimizes the encoding of motion information by MT neurons, with a behavioural impact of optimizing information in pursuit eye movements, minimizing visual tracking errors and thereby improving vision of moving objects. Pursuit behaviour arises from a population of MT neurons^43^. One could imagine that a sensory population could have optimal sensitivity to motion fluctuations when individual units do not. As it happens in the pursuit system, and perhaps generally throughout sensory cortex, single neurons optimize gain individually. Determining the impact of single-neuron gain changes on population-level motion estimates will require large-scale simultaneous recordings of the MT population, to measure the structure of signal- and noise-driven correlations.

Two very different mechanisms have been proposed to explain gain adaptation to velocity variance in fly H1 (refs 5, 6). Bialek and colleagues^5^,^6^,^35^ described the effect as adaptation, meaning a rescaling of the system's representation of visual motion signals. Borst *et al*.^39^ and Sompolinsky and colleagues^40^ proposed that a similar phenomenon could be elicited without a state change from a correlation-based (Reichardt) motion detector with a saturating nonlinearity at high frequencies. As variance in the stimulus increases, the high-frequency response saturates sooner than the low-frequency response, creating an apparent drop in gain without any actual change in the system parameters. Although subsequent work ultimately supported the adaptation hypothesis, based on the failure of the static model to predict the mixture of adaptation timescales observed in the fly, retina and cortical slice recordings^13^,^14^,^15^,^55^, the static nonlinearity mechanism remains an interesting possibility. The nature of recording from behaving monkeys makes the identification of long adaptation timescales quite difficult. Although cortical slice experiments could use long sequences of variance changes in injected current over many minutes, we were constrained by the monkey's ability to maintain fixation and we were unable to resolve differences in adaptation dynamics as a function the duration of stimulus presentation^13^. However, two features of results argue for adaptation over a saturating nonlinearity model. First, we did not observe saturation in either MT or pursuit responses (Figs 2 and 3). Second, we found that reducing the high-frequency content of our stimulus to match pursuit's frequency response preserved gain rescaling (Fig. 3). We note that Bair and Movshon^56^ did observe changes in MT neuron responses that were consistent with a static nonlinearity model, but they manipulated temporal frequency content of the stimulus rather than variance, and so our results are not directly comparable.

Several classes of mechanisms have been proposed to account for gain adaptation in other systems, including modulation of channel conductances, synaptic facilitation/depression and circuit effects. Intrinsic conductance changes have been implicated in gain adaptation occurring on seconds-long timescales. For example, sodium channel inactivation in salamander retinal ganglion cells^8^, modulation of a slow Ca^2+^-sensitive K^+^ after-hyperpolarization conductance in barrel cortex^37^,^57^ and the balance of sodium and potassium currents in mouse sensorimotor cortex^18^ have been implicated in adaptation to input variance. In each of these systems, the timescales of adaptation are substantially longer than the 40–70 ms timescale we observe in the primate.

Information flow in thalamocortical and corticocortical pathways is gated by adapting metabotropic and ionotropic glutametergic synapses that facilitate or depress respectively, modulating the response gain of their targets. In the visual system, Scanziani and colleagues^58^ demonstrated gain modulation of LGN activity within ∼50 ms by V1 layer 6 cortical projecting neurons. The reported timescale of thalamocortical and corticocortical synaptic facilitation/depression is ∼30-100 ms^47^, very similar to to the timescale we measured. While synaptic gain changes alone are typically associated with large changes in firing rate^58^ which we did not observe, recent studies have identified network effects that might produce rapid gain changes without affecting average firing rate^59^. For example, balanced barrages of excitatory and inhibitory synaptic activity rapidly increase neuronal responsiveness on the timescale of tens of ms^60^. Interaction between local recurrent circuit activity and non-linear dendritic properties has also been proposed as a possible mechanism for cortical gain adaptation that may operate on the fast timescales we observe^61^,^62^. Recurrent activity among similarly tuned neurons could regulate response gain, amplifying the response to thalamic input as well as sharpening the response selectivity or increasing signal-to-noise ratio^62^,^63^,^64^ which might account for the information maximization we observe in individual MT neurons.

Given the diversity of adaptive mechanisms available to neural systems, it seems likely that most if not all sensory systems have the capacity to adaptively encode the stimulus features to which they are most sensitive^2^,^65^,^66^. This study demonstrates that the impact of adaptive coding reaches beyond information representation of single neurons to the performance of behavior. On longer timescales, the brain has the ability surpass the limits of optimal sensory coding by building experience-based models of the world^67^,^68^,^69^,^70^,^71^ that allow for predictive neural responses^72^,^73^, anticipatory behaviours^74^,^75^,^76^,^77^,^78^ and motor learning^79^,^80^,^81^. The next challenge will be to determine how neurons balance the benefits of efficient sensory representation with other constraints^82^,^83^ such as prediction in guiding behaviour.

## Methods

Eye movement recordings and extracellular recordings from extrastriate cortical area MT/V5 were made in two adult male rhesus monkeys (*Macaca mulatta*); a third monkey participated in behavioural experiments only. Animals were implanted with a scleral coil in one eye, a post for head restraint and a recording chamber using sterile surgical technique under anaesthesia. All surgical and experimental procedures were approved in advance by the University of Chicago's Institutional Animal Care and Use Committee and were in strict compliance with the US National Institutes of Health Guide for the Care and Use of Laboratory Animals. We trained animals in basic pursuit tasks before collecting these data. The animals viewed bright targets against the dark screen of a Sony GDMFW950 fast CRT display (100–120 fps, 1,024 × 768 pixels) in a dimly lit room. Eye movements were sampled every millisecond, filtered and digitized for future analysis^45^. Experiments were organized into trials lasting 2–3 s. Animals were rewarded at the end of a trial for keeping the eye within several degrees of the target during specified periods. For pursuit tasks, animals were required to maintain fixation within 2° of a stationary fixation spot at trial onset and to be within 3° of the target during the final 200 ms of pursuit. Gaze accuracy was not penalized during time windows used for data analysis. During physiology experiments, animals had to maintain fixation within 2° throughout the trial.

Horizontal and vertical eye positions were sampled at 1 ms intervals, low-pass filtered and differentiated. The velocity components were translated into instantaneous eye direction, to allow comparison of stimulus and response in the same units (degrees). Each trial was inspected and trials with blinks or saccades during the motion interval were discarded from further analysis.

Magnetic resonance imagings of the monkeys were obtained before implantation to guide chamber location. We recorded from visual cortex with an array of three quartz-platinum/tungsten electrodes (TREC, Germany). We localized area MT based on stereotactic coordinates, receptive field size, motion selectivity and other physiological response properties in MT and in surrounding structures. We sampled neural activity at 30 kHz (Plexon Omniplex) and stored waveforms for offline spike sorting. We performed online analyses to map the direction and speed tuning, and the size and location of each unit's excitatory receptive field. We identified single units through principal component analysis of spike waveforms in tandem with inspection of interspike interval distributions.

### Visual stimuli

Stimuli consisted of random dot patterns (2 dots deg^−2^) that moved in an aperture against the dark background of the monitor. In physiology experiments, the dots moved within a stationary aperture, while the monkey maintained fixation, but in behavioural experiments both the pattern and aperture (4°) translated across the screen at a constant speed. Dots moved coherently such that the direction and speed of each dot was identical at each time step, but the pattern direction had an added stochastic perturbation that was updated every 20 ms (two frames) from uniform distributions with different variances. Target speeds were 20–25° s^−1^ for pursuit and were typically set to the preferred speed of each MT unit (2–96° s^−1^, mean=29° s^−1^). Some pursuit experiments used 0.25° spot targets with identical motion statistics. Trials were often configured to contain one or more steps in direction variance at fixed times within the trial.

### Receptive field mapping

Visual stimuli were tailored for each neuron to span the classical receptive field and to fall on particular portions of the direction tuning curve. We mapped tuning curves with full-field patterns (56° by 38°) whose direction spanned the circle with 15° spacing and plotted the tuning curve. We then determined the speed tuning curve using preferred direction motion and log_2_ speed spacing. Receptive fields were localized using 2–5° patterns that appeared in different spatial locations. We selected a centre direction for the fluctuation stimuli based on each unit's direction tuning curve, testing on one or both flanks. Values for the size of our sample (*n*) represent the number of experiments rather than the number of neurons. We recorded from a total of 44 MT neurons (*n*=26 monkey 1; 18 monkey 2) for this study.

### Linear fitting

We fit linear relationships between input (stimulus direction) and output (spike count or eye direction), to define the response gain (see Figs 2 and 3). We used principal component analysis to determine the dominant mode of variation in the data sample by minimizing the summed perpendicular distance between the data points and the fit.

### Mutual information estimates

We used the direct method to compute the mutual information between stimulus and response^41^. We divided the trial into overlapping time windows of 60 ms. In each time window, *T*, we adaptively binned the values of stimulus, *θ(T)*, and response (either spike count *n(T)* or the eye direction *θ*_E_*(T)*) such that equal numbers of examples occurred in each bin. We then formed the joint probability distribution between the stimulus and response, for example, P(*n(T)*, *θ(T−τ)*) for neurons or P*(θ*_E_*(T)*, *θ(T−τ))* for pursuit for each time delay, *τ.* Information values peaked at a delay equal to the response latency, which was somewhat stimulus-variance dependent. Simplifying the notation to P_*T*_*(n, θ)*, the mutual information is defined as


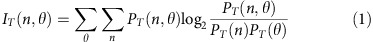


where *I*_*T*_*(n, θ)* quantifies in bits the amount of information that a single observation of a spike count of *n* in the time window *T* provides about the direction of motion. *P*_*T*_*(n)* is the total probability of observing *n* spikes after counting over the time interval, *T*, averaged over all stimuli. In our case, all stimuli occurred with roughly equal probability, *P*_*T*_*(θ).* The equation is identical for computing information from eye movements, exchanging *P*_*T*_*(θ*_E,_*θ)* for *P*_*T*_*(n,θ)* and summing over the number of bins (20) used to discretize the eye direction.

### Finite sample bias correction

We used a procedure to minimize the effects of finite sample size on our estimates of information, following the methods of refs 42, 43. By randomly drawing different numbers of samples (*N*) from our total trial set for each neuron (or pursuit dataset), we looked for the expected systematic behaviour as follows:


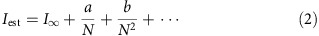


and extracted *I*_∞_ as our best estimate. The number of repeats in our data set gave reasonable linear behaviour keeping first-order terms in *N* only. It is noteworthy that the extrapolated estimate of information for an infinite data set is always smaller than the value measured from a finite data set.

### Change point detection

To quantify adaptation dynamics from the spike trains themselves, we used a log-likelihood method. We time shifted the responses by the average latency, found the total spike count or time-averaged eye direction and the stimulus direction in successive 20 ms time windows. We pooled windows over each motion segment to measure the joint distribution of binned counts and target directions, *P(r, θ)*, or binned eye and target directions, *P(θ*_E_*, θ*_T_), for low- and high-variance conditions. We then stepped through the response on each trial and computed the cumulative likelihood that the series of response values came from a low-variance or high-variance stimulus condition. We defined the cumulative likelihood at time *T*, *C(T)*, for an HTL variance step trial as


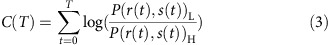


where *r(t)* represents the response in time window *t*, *s(t)* the stimulus in the same time window, the subscript *L* indicates a low-variance condition and *H* represents a high-variance condition. For each data set, we defined a threshold from the s.d. of *C(T)* over all time steps (and all trials) before the variance shift. We then started integrating the likelihood from the time of the shift and computed the cumulative likelihood over time for each trial^48^. Negative *C*-values were reset to 0. We defined the change point as the time at which *C* exceeded the threshold.

### Data availability

The data that support the findings of this study are available from the corresponding author upon request.

## Additional information

**How to cite this article:** Liu, B. *et al*. Efficient sensory cortical coding optimizes pursuit eye movements. *Nat. Commun.* 7:12759 doi: 10.1038/ncomms12759 (2016).
